# Effects of a Massive Open Online Course on osteoarthritis knowledge and pain self-efficacy in people with hip and/or knee osteoarthritis: protocol for the MOOC-OA randomised controlled trial

**DOI:** 10.1186/s12891-023-06467-x

**Published:** 2023-05-15

**Authors:** Rachel K. Nelligan, Rana S. Hinman, Thorlene Egerton, Maya Gregory, Neil Bidgood, Ms Fiona McManus, Anurika P. De Silva, Karen E. Lamb, Kim L. Bennell

**Affiliations:** 1 Centre for Health, Exercise and Sports Medicine, Department of Physiotherapy, School of Health Sciences, The University of Melbourne, Melbourne, VIC Australia; 2 Community-Based Consumer Representative, Person With Knee Osteoarthritis, Melbourne, Australia; 3 Centre for Epidemiology and Biostatistics, Melbourne School of Population and Global Health, University of Melbourne, Melbourne, VIC Australia

**Keywords:** Osteoarthritis, Patient education, RCT, Trial, E-learning, Knowledge, Self-efficacy

## Abstract

**Background:**

Osteoarthritis (OA) is a prevalent, chronic joint condition that commonly affects the knee and hip causing pain, impaired function, and reduced quality of life. As there is no cure, the main goal of treatment is to alleviate symptoms via ongoing self-management predominantly consisting of exercise and weight loss (if indicated). However, many people with OA do not feel adequately informed about their condition and management options to self-manage effectively. Patient education is recommended by all OA Clinical Practice Guidelines to support appropriate self-management, but little is known about the optimal delivery method and content. Massive Open Online Courses (MOOCs) are free, interactive, e-learning courses. They have been used to deliver patient education in other chronic health conditions but have not been used in OA.

**Methods:**

A two-arm parallel-design, assessor- and participant-blinded superiority randomised controlled trial. People with persistent knee/hip pain consistent with a clinical diagnosis of knee/hip OA (*n* = 120) are being recruited from the Australia-wide community. Participants are randomly allocated into one of two groups i) electronic information pamphlet (control group) or ii) MOOC (experimental group). Those allocated to the control group receive access to an electronic pamphlet about OA and its recommended management, currently available from a reputable consumer organisation. Those allocated to the MOOC receive access to a 4-week 4-module interactive consumer-facing e-Learning course about OA and its recommended management. Course design was informed by behaviour theory and learning science, and consumer preferences. The two primary outcomes are OA knowledge and pain self-efficacy with a primary endpoint of 5 weeks and a secondary endpoint of 13 weeks. Secondary outcomes include measures of fear of movement, exercise self-efficacy, illness perceptions, OA management and health professional care seeking intentions, physical activity levels, and actual use of physical activity/exercise and weight loss, pain medication, and health professional care seeking to manage joint symptoms. Clinical outcomes and process measures are also collected.

**Discussion:**

Findings will determine whether a comprehensive consumer-facing MOOC improves OA knowledge and confidence to self-manage joint pain compared to a currently available electronic OA information pamphlet.

**Trial registration:**

Prospectively registered (Australian New Zealand Clinical Trials Registry ID: ACTRN12622001490763).

## Background

Osteoarthritis (OA) is a prevalent and chronic joint condition that commonly presents in the knee and hip causing joint pain, impaired function, and reduced quality of life [1]. As there is currently no cure, the main goal of treatment is to alleviate symptoms via ongoing self-management using first-line approaches of exercise and weight loss, if indicated [2]. Joint replacement surgery, which is invasive, costly and can result in serious adverse events [3], should only be considered as a last resort for those with severe symptoms who have not seen improvement from recommended non-surgical approaches. However evidence suggests that less than 1 in 3 of those referred to an orthopaedic surgeon feel informed about how to self-manage [4] and the majority are dissatisfied with the quality and amount of information they received about OA and its recommended management [5]. People with OA want more information about their condition and its management options so that they can ‘take action’ to improve their own health state and quality of life [5].

Although patient education is advocated in all OA Clinical Practice Guidelines as essential to support appropriate self-management [2], the optimal delivery method and content remain uncertain. OA patient education interventions evaluated in trials have been found to lack comprehensiveness, not be based on best evidence, behaviour theory or learning science principles, nor designed in collaboration with people with OA [6]. Massive Open Online Courses (MOOCs) are free, interactive, e-learning courses that are used to deliver university-quality education. They have many benefits including convenience, features that support pedagogical principles to enhance learning, are easily updated and allow unlimited registrants so are infinitely scalable [7]. Although limited, there is evidence suggesting MOOCs may be an effective method of providing patient education in Type 2 diabetes [8] and in dementia risk reduction [9]. Thus, MOOCs could be a scalable and effective method of providing comprehensive education to people with OA about the condition and its recommended management practices.

Our team has developed the first consumer-facing MOOC about OA and its management, for people with OA, which we aim to evaluate in this trial. Our primary hypothesis is that people with hip and/or knee OA who receive the 4-module 4-week MOOC will have greater improvements in OA knowledge and/or self-efficacy for pain at 5 weeks compared to those who receive a typical OA education intervention: an electronic OA information pamphlet available from a reputable consumer organisation. Our secondary hypothesis is that those receiving the MOOC will have more favourable changes in a range of other outcomes including fear of movement, exercise self-efficacy, illness perceptions, OA management and health professional care seeking intentions, physical activity levels, and actual use of physical activity/exercise and weight loss, pain medication, and health professional care seeking to manage joint symptoms.

## Methods/design

### Trial design

This is a two arm, parallel group, superiority randomised controlled trial (RCT) reported according to SPIRIT guidelines [10] and TIDieR [11].

### Participants

People with persistent knee/hip pain consistent with a clinical diagnosis of knee/hip OA [12] are currently being recruited from the Australian-wide community via advertisements (e.g., print/radio/social media) and our volunteer database. Eligibility criteria are presented in Table 1. Informed consent is obtained prior to baseline questionnaires from all participants via online forms using REDCap™ (Research Electronic Data Capture) hosted at the University of Melbourne [13, 14]. Ethics approval was obtained.

**Table 1:** Eligibility criteria

Inclusion criteria	Exclusion criteria
i. live in Australia; ii. have an unreplaced (native) hip or knee joint that meets the National Institute for Health and Care Excellence clinical criteria for OA: [12] a. aged 45 years or over; b. activity-related pain at the joint; c. joint morning stiffness that lasts ≤ 30 min or no morning stiffness at the joint iii. history of pain at the joint for ≥ 3mths; and iv. joint pain on most days of the past month; v. have access to a computer with internet connection and an email address; and vi. able to give informed consent and willing to commit to all study evaluation and assessment procedures	i. self-reported systemic arthritis (e.g., rheumatoid arthritis, gout); ii. scheduled for lower limb joint surgery in the next 13 weeks; iii. completed an online education course about OA that involved at least 2 h of learning in total in the past 12 months; and/or iv. unable to easily read and understand English

### Procedures

**Fig. 1:**
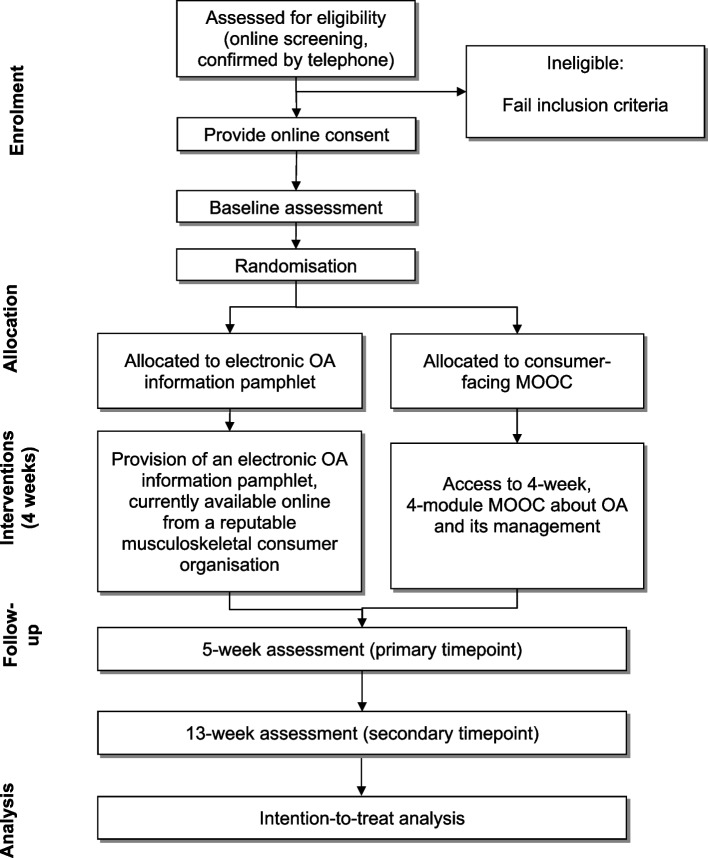
Flow diagram of trial procedures

Figure 1 outlines trial phases. Participants first complete eligibility screening via an online survey (REDCap™). The survey outlines trial details and participant requirements and contains questions to assess inclusion criteria. Potentially eligible people are telephoned by a researcher who provides a verbal description of the study and reviews and confirms inclusion and exclusion criteria. Eligible participants are then sent a link to an online consent form (including the trial’s plain language statement) via email. Those who complete the consent form receive a link to the online baseline questionnaire. In cases where multiple joints are equally symptomatic, the participant is asked to select one hip/knee as the focus of assessment.

### Randomisation and blinding

Participants are enrolled into the study on completion of baseline questionnaires and are randomised (1:1 ratio) into one of two groups i) electronic OA information pamphlet (control group) or ii) MOOC (experimental group), using randomly permuted blocks of varying sizes, stratified by eligible joint (hip or knee). The randomisation list was computer-generated by an independent statistician and is managed by a researcher centrally to ensure concealment. Participants are blinded to group allocation using limited disclosure (told that the study is investigating two different types of information material about OA but without explicit detail about either intervention nor the study hypotheses given). As primary outcomes are participant-reported, and participants are blinded, by default the assessors of these outcomes are blinded. Statistical analyses will be performed while blinded.

### Interventions

#### Electronic OA information pamphlet (control group)

Participants in this group receive access to an electronic information pamphlet about OA and its management, currently available online from a reputable consumer organisation, Musculoskeletal Australia. Immediately after randomisation, participants receive an email from the research team containing a link to a PDF version of the pamphlet (mskweb.msk.org.au/$web/2022/07/Osteoarthritis.pdf) and are asked to read the pamphlet within the coming 5 weeks. On completion of their involvement in the study (after 13-week assessment is completed), they are provided access to the experimental group intervention (MOOC).

#### Consumer-facing Massive Open Online Course (experimental group)

Participants in this group receive access to the 4-week 4-module consumer MOOC which is housed on FutureLearn, a web-based platform for university-quality online education courses. Immediately after randomisation, participants receive an email from the research team containing a link to the MOOC registration page (futurelearn.com/courses/taking-control-hip-and-knee-osteoarthritis). They are instructed to register for the free version of the course and complete Module 1 within 7 days. Thereafter, they are encouraged to complete the remaining three modules following the course schedule (modules are ‘unlocked’ week-by-week).

**Fig. 2:**
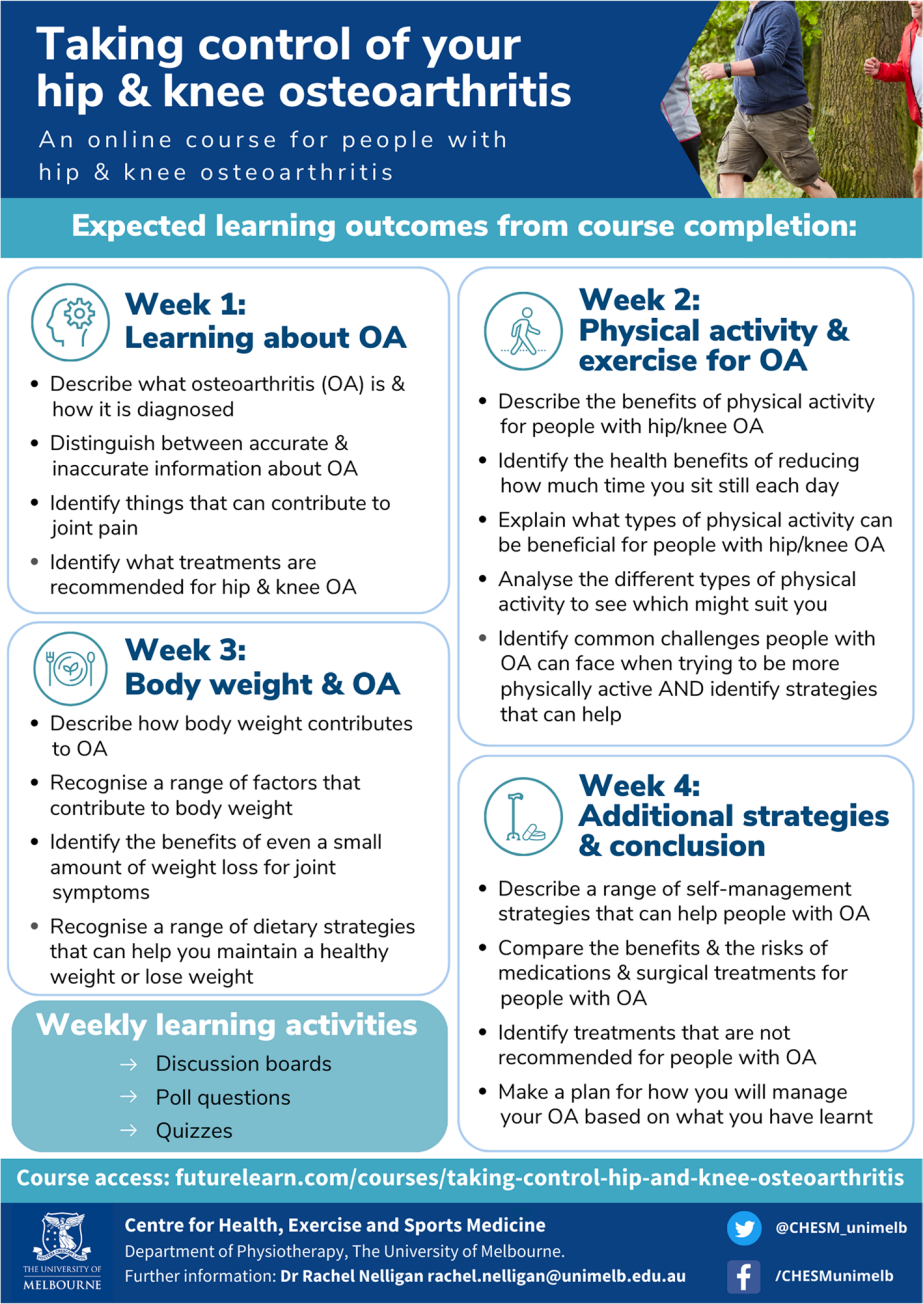
Learning outcomes per online course module

Figure 2 presents the course learning outcomes per module. Content is presented over four modules: (1) Learning about OA; (2) Physical activity and exercise for OA; (3) Body weight and OA; (4) Additional management strategies and conclusion. Each module includes a range of learning activities including non-moderated discussion boards for learner interaction, poll questions, quizzes aligned with learning outcomes and downloadable documents to facilitate turning learnings into action (e.g., physical activity logbooks, meal portion size guide, self-management action plan). Each module also contains a ‘finding out more’ section which includes links to external credible resources, information about a range of healthcare professionals who may be able to provide further assistance, and references to scientific papers that support content. The time required to complete all four modules and learning activities is approximately four hours (about one hour per module/week), although total duration depends on the user’s interaction with the provided resources, external links, and references.

The course was designed by the research team (RKN, KLB, RSH) and a consumer MOOC review team (5 people with hip and/or knee OA). In addition, the course was evaluated by FutureLearn’s Editorial Team to ensure that the course met quality assurance standards for accessibility and pedagogy-informed design. Course design was underpinned by four key pillars that are recommended for the development of high-quality OA education [6]:

##### Pillar 1. Based on previous OA research

Course content was based on evidence-based Clinical Practice Guidelines [2, 12]; key patient messages about OA and its management [15]; and core capabilities for the delivery of optimal OA care [16].

##### Pillar 2. Informed by behaviour theory

The Common-Sense Model of Self-Regulation [17] guided the approach for addressing illness perceptions that can influence treatment/management choices. The model also informed lesson content addressing maladaptive beliefs about identity, and the causes, consequences, controllability, and prognosis of OA. Social learning and self-efficacy theory [18] informed behaviour change lesson content aimed at empowering participants to make appropriate management choices and enact effective self-management behaviours. Lesson content incorporated the behaviour change techniques of goal setting, problem solving, action planning, self-monitoring, verbal persuasion about capability, instruction on how to perform the behaviour, credible source, and demonstration of behaviours/role modelling [19].

##### Pillar 3. Informed by learning science

The overall course design was underpinned by a pedagogy of social learning, the approach applied by FutureLearn based on their extensive experience and expertise in delivering online education. Fundamental to this approach is learning through storytelling and interaction with others. In addition, learning science principles were applied to course components, including Bloom's taxonomy, Mayer's principles of multimedia design, and immediate and elaborated feedback [20].

##### Pillar 4. Incorporates consumer involvement

Consumer involvement was incorporated via online survey completed by 348 people with hip and/or knee OA from our Centre’s consumer Knowledge Translation Network. The survey gauged interest in a consumer-facing online course and collected feedback on the proposed content and learning outcomes. From this, we identified that 99% were interested in the concept and 100% thought the proposed content would be useful for them. Once a prototype was developed, extensive feedback was then collected from our consumer MOOC review team using an iterative think aloud approach [21]. This involved each member of the MOOC review team independently completing each course module while being observed by a member of the research team (RKN) via zoom screen share. Based on feedback, refinements were made to the course after each individual think aloud session, ultimately resulting in the final version of the course. Appendix 1 provides an overview of refinements made.

### Outcomes

Table 2 lists all baseline descriptive data and outcome measures. All assessments (baseline, 5-week [primary] and 13-week [secondary]) are completed remotely via online questionnaires (REDCap™). Participants completing both 5- and 13-week questionnaires are given a $AUD50 gift voucher in gratitude for their participation.

**Table 2:**
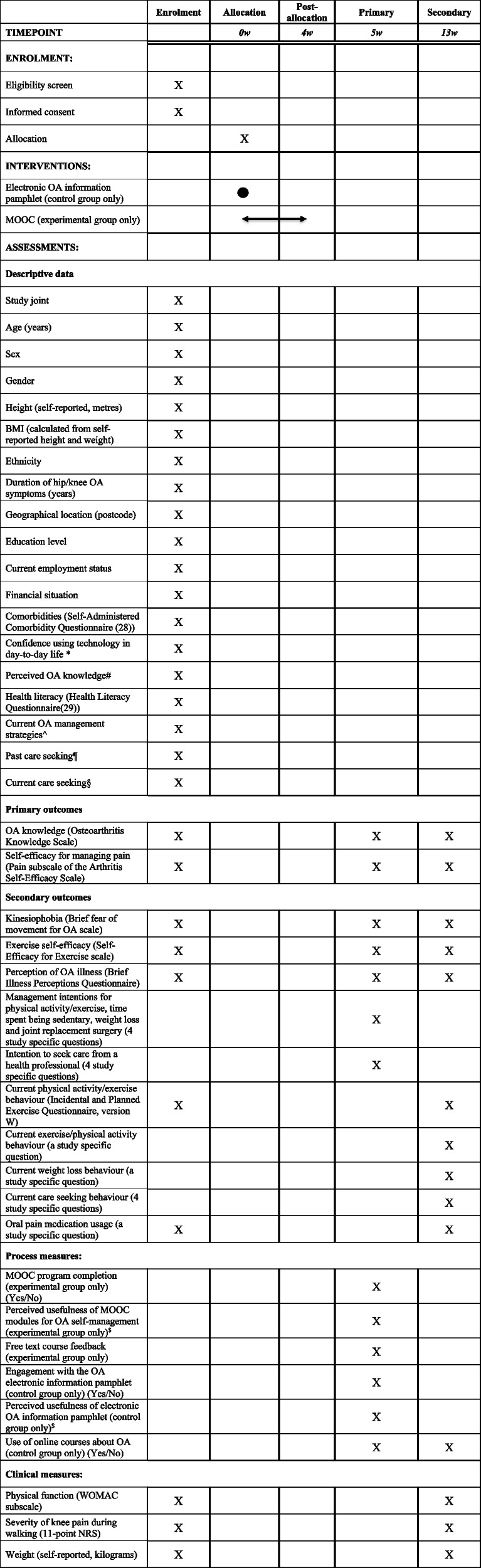
Schedule of enrolment, intervention, and assessments [22, 23]

*MOOC* Massive Open Online Course, *WOMAC* Western Ontario and McMaster Universities Osteoarthritis Index, *BMI* Body Mass Index, *OA* osteoarthritis, *NRS* Numeric rating scale

^*^via 4-point Likert scale with terminal descriptors 1 = not at all confident; 4 = extremely confident

^#^via a 4-point Likert with response options none, a little, some, a lot

^via Yes/No response to both “For your osteoarthritis, are you currently making efforts to i) lose weight (e.g. dietary changes) ii) increase the amount/intensity of physical activity/exercise you do?”

^¶^Yes/No response to “Have you ever sought care for your knee or hip pain from any health professional before?”

^§^Yes/No response to “In the past month, have you seen a health professional for advice about your osteoarthritis?” Those selecting “Yes” will be asked which health professionals they have seen

^$^via 4-point Likert scale with response options: 1 = not at all useful; 2 = slightly useful; 3 = moderately useful; 4 = extremely useful. Dichotomised into useful (slightly, moderate, extremely) and not useful (not at all useful)

#### Primary outcomes

The two primary outcomes are self-reported measures collected at baseline, 5 and 13 weeks.

##### OA knowledge

The Knee/Hip Osteoarthritis Knowledge Scale (OAKS) [24] includes 11-items that measure knowledge about hip/knee OA, including causation, diagnosis, symptom interpretation, management principles, treatment and self-care options. Each item is rated using a 5-point Likert scale (response options: False; Possibly False; Unsure; Possibly True; True). Items 1–4, 7, and 11 are scored in reverse. All item scores are added for a total score range of 11 to 55. Higher scores indicate more accurate knowledge. This measure has acceptable psychometric properties when used in mixed cohorts of people with hip and knee OA [25].

##### Pain self-efficacy

The Pain subscale of the Arthritis Self-Efficacy Scale (ASES pain) [26] includes 5 items that measure one’s self-efficacy for managing their osteoarthritis pain. Each item is rated using a 10-point scale (1 = very uncertain and 10 = very certain). Scores are the mean of all items for a total score range of 1 to 10. Higher scores indicate greater pain self-efficacy. This measure has established validity, reliability and responsiveness in OA [26].

#### Secondary outcome measures

A range of self-reported secondary outcomes are measured at baseline, 5- and 13-weeks, unless indicated otherwise.

*Kinesiophobia* via the 6-item Brief Fear of Movement for OA Scale [25]. Each item is rated using a 4-point Likert scale from 1 = Strongly disagree to 4 = Strongly agree. All item scores are added for a total score range of 6 (minimal fear) to 24 (maximal fear).

*Exercise self-efficacy* via the 9-item Self-efficacy for Exercise (SEE) Scale [27]. Each item is rated on an 11-point scale. Scores range from 0–90 with higher scores indicating higher self-efficacy for exercise.

*OA illness perceptions* via 8-items of the Brief Illness Perceptions Questionnaire (B-IPQ) [28]. Each item is scored on a NRS (0 to 10). Items 3, 4, and 7 are scored in reverse. All item scores are added for a total score range of 0–80. Higher scores represent a more threatening view of OA.

*Management intentions* (week 5 only) via four study specific items. Participants are asked about their intentions over the next 2 months to 1) increase amount/intensity of physical activity/exercise; 2) reduce the amount of time spent sedentary; 3) make efforts to lose weight; and 4) have hip/knee joint replacement surgery in the next 2 years. Responses options are Yes/No.

*Intention to seek care from a health professional* (week 5 only) via four study specific items. Participants are asked about their intentions to seek care in the following 2 months for 1) weight loss; 2) an exercise/physical activity program; 3) pain relieving medication; and 4) joint replacement surgery. Responses options are Yes/No. For each item ‘Yes’ is selected, participant will be asked to select which health professionals they intend to see.

*Current physical activity* (baseline and week 13): via the 10-item Incidental and Planned Exercise Questionnaire, version W (IPEQ-W) [29]. Scores range from 0–128 with higher scores indicating higher levels of activity.

*Current exercise/physical activity behaviour* (week 13 only): via the question “Over the past 2 weeks, how would you compare your amount of physical activity/exercise to when you started the study?”, rated on a 3 point-Likert with response options less/same/more.

*Current weight loss behaviour* (week 13 only): via the question “In the past 2 weeks, did you make any effort to lose weight (e.g. diet changes)?” with response options Yes/No.

*Current care seeking behaviour* (week 13 only): via 4 study specific items. Participants are asked if they have consulted a health professional since they enrolled in the study to discuss 1) weight loss; 2) an exercise/physical activity program; 3) pain relieving medication; and 4) joint replacement surgery. Responses options are Yes/No. For each item ‘Yes’ is selected, participant will be asked to select which health professionals they intend to see.

*Oral pain medication usage in the prior month* (baseline and week 13) via self-reported use of common oral pain-relieving medications taken at least once a week in the prior month for knee/hip pain. Participants are asked to select Yes/No from options: 1) oral non-steroidal anti-inflammatory drugs; 2) analgesics (paracetamol combinations); 3) oral corticosteroids; and 4) oral opioids.

#### Other measures

##### Clinical measures

(Baseline and week 13) 1) average severity of knee pain during walking in the past week is measured via an 11-point NRS (0 = no pain and 10 = worst pain possible); 2) physical function is measured via the 17-item Western Ontario and McMaster Universities Osteoarthritis Index (WOMAC) physical function subscale (range 0 to 68, higher scores indicating higher dysfunction) [30]; and 3) body weight (self-reported in kilograms).

##### Process measures

Several process measures are collected at 5 weeks regarding engagement with and perceived usefulness of each allocated resource (i.e. MOOC or electronic OA information pamphlet). Table 2 lists all process measures.

### Data analysis, monitoring and auditing

#### Sample size calculation

A sample size of 60 participants per arm (120 in total) is required for 90% power to demonstrate that the consumer-facing MOOC is superior to the control with a two-sided 2.5% significance level (accounting for multiple comparisons across the two primary outcomes by using Bonferroni correction) and allowing for a 20% dropout rate. The sample size calculation was based on the following assumptions: a standardised between-group effect size of 0.625 for pain self-efficacy (based on our prior research [31], corresponding to an absolute between-group difference in mean change from baseline to 5 weeks of 1 unit in ASES pain subscale score favouring the MOOC, with within-group standard deviation (SD) of 1.6 units [31], correlation between measures across all three timepoints of 0.5 (i.e., compound symmetry variance–covariance matrix) [31], and using a constrained longitudinal data analysis (cLDA) model [32]. With this sample size, we also have at least 90% power to detect a between-group effect size of 0.8 for OA knowledge (conservative for this type of program [33]), corresponding to an absolute between-group difference in mean change from baseline to 5 weeks of 4.6 units in KOAKS/HOAKS score favouring the MOOC, with within-group SD of 5.8 units [31], and correlation between measures across all three timepoints of 0.2 [31].

#### Data analysis

The biostatisticians (FM, KEL, ADS) will devise a statistical analysis plan for the study prior to being unblinded to group allocation. It will be published on our Centre’s website. Analyses will include all participants according to their group allocation (intention-to-treat). Each primary outcome will be analysed using a cLDA [32] model. The response will consist of all KOAKS/HOAKS or ASES pain scores (at baseline, 5 and 13 weeks), and the model will include factors for group, time (categorical), and group-by-time interaction, with the restriction of a common baseline mean across treatment groups. The mean change in KOAKS/HOAKS or ASES pain scores from baseline to each follow-up timepoint between the groups will be obtained. The primary hypothesis will be evaluated by obtaining the estimated differences between groups in mean change in KOAKS/HOAKS and ASES pain score from baseline to 5 weeks post randomisation, and multiplicity adjusted two-sided 95% confidence intervals and *p*-values. These models provide valid inference in the presence of missing data if the data are missing at random (MAR). An analysis will be conducted using the delta-adjustment method under the pattern-mixture modelling framework in the context of multiple imputation to assess sensitivity to missingness not at random. Secondary outcomes: Management intentions and care seeking intentions at 5 weeks, and care seeking behaviour, exercise and weight loss behaviours and pain medication usage (adjusted for baseline usage) at 13 weeks will be analysed using log-binomial regression models. Physical activity levels at 13 weeks will be analysed using a linear regression model adjusted for baseline physical activity. Other continuous outcomes will be analysed the same as the primary outcomes. All analysis models will be adjusted for the stratification factor, eligible joint (hip/knee). Process measures and other baseline measures will be summarised using frequency (proportion) for binary measures and mean (SD)/ median (inter-quartile range) for continuous measures.

#### Monitoring

The research team meet fortnightly to review recruitment and monitor trial progress.

### Patient and public involvement

As described earlier, 348 people with hip/knee OA were involved in the MOOC design via an online survey to gauge interest in the intervention concept (MOOC) and its proposed content. Additioanlly, a MOOC review team (5 people with hip/knee OA) provided feedback (totalling > 21 h) on versions of the MOOC using an iterative think aloud process. One person with OA (NB) is an investigator on this study. They provided input into the trial design and processes including reviewing participant-facing materials (e.g., plain language statement and consent form). They will assist with interpretation of findings.

### Dissemination plans

Findings will be disseminated via conference presentations; journal publications; lay summaries to participants and via our Centre’s social media channels and Knowledge Translation Network. Findings will also be disseminated by the Physiotherapy Research Foundation’s channels.

## Discussion

This RCT will determine whether a comprehensive consumer-facing eLearning course (MOOC) about OA and its management improves OA knowledge and/or confidence to self-manage symptoms compared with a typical OA education intervention: an electronic OA information pamphlet available from a reputable consumer organisation.

## Data Availability

Not applicable.

## Appendix 1

### Summary of course refinements based on feedback from the consumer think aloud evaluations

1. Minor changes to the course including:
  - the reordering of content within modules/weeks 1 and 2;
  - changes to text to improve readability, all modules/weeks;
  - modifications to end of module quiz questions, module/week 1.
2. One major change:
  - the redesign of module 3 “Body weight and osteoarthritis”. During review, participants indicated that the original module focused too heavily on the negative impacts of body weight on joint and overall health. It was thought that this could evoked feelings of guilt and judgment and may not be relevant to all people (e.g., those who have low body weight or those with low interest in the topic). Based on participants feedback the module was re-designed to remove content describing the negative impacts of high body weight and instead was written to educate about the positive benefits of healthy weight maintenance and weight loss.

## Abbreviations

MOOC: Massive Open Online Course
RCT: Randomised controlled trial
